# Anesthetic management of a patient with mandibular hypoplasia, deafness, progeroid features, lipodystrophy syndrome: a case report

**DOI:** 10.1186/s40981-024-00747-8

**Published:** 2024-10-10

**Authors:** Ryo Sekiguchi, Michiko Kinoshita, Yoko Sakai, Katsuya Tanaka

**Affiliations:** 1 Department of Anesthesiology, Institute of Biomedical Sciences, Tokushima University Graduate School, 3-8-15 Kuramoto-Cho, Tokushima-Shi, Tokushima, 770-8503 Japan; 2 Department of Anesthesiology, Tokushima University Hospital, Tokushima, 2-50-1 Kuramoto-Cho, Tokushima-Shi, Tokushima, 770-8503 Japan; 3 Division of Anesthesiology, Tokushima University Hospital, 2-50-1 Kuramoto-Cho, Tokushima-Shi, Tokushima, 770-8503 Japan

**Keywords:** Case report, General anesthesia, Lipodystrophy, Mandibular hypoplasia, MDPL syndrome, Progeroid syndrome

## Abstract

**Background:**

Mandibular hypoplasia, deafness, progeroid features, and lipodystrophy (MDPL) syndrome is a rare autosomal dominant disorder that presents unique challenges for anesthetic management due to its multisystemic manifestations. This report outlines the anesthetic considerations for MDPL patients based on our case experience.

**Case presentation:**

A 15-year-old male with MDPL syndrome underwent testicular extraction under general anesthesia. Insertion of a peripheral venous catheter was challenging due to scleroderma-like skin. Although the facial features of MDPL syndrome suggested a difficult airway, intubation with a McGrath™ Mac video laryngoscope was successful. Despite MDPL syndrome’s association with hypertriglyceridemia due to lipodystrophy, this patient’s triglyceride levels were normal. Thiamylal and sevoflurane were used without issues such as delayed emergence from anesthesia.

**Conclusions:**

MDPL syndrome requires careful preoperative assessment and tailored anesthetic management due to potential airway challenges arising from its distinctive facial features and the possibility of altered anesthetic pharmacokinetics associated with lipodystrophy.

**Supplementary Information:**

The online version contains supplementary material available at 10.1186/s40981-024-00747-8.

## Background

Mandibular hypoplasia, deafness, progeroid features, and lipodystrophy (MDPL) syndrome is a rare autosomal dominant genetic disorder first described by Shastry et al. in 2010 [1]. In 2013, Weedon et al. identified heterozygous mutations in the POLD1 gene as the cause of MDPL syndrome [2]. As of 2024, fewer than 40 cases have been reported worldwide, highlighting its rarity [3].

MDPL syndrome is a progeroid disorder characterized by a constellation of clinical features, including a prominent loss of subcutaneous fat, distinctive facial characteristics, and sensorineural deafness. Patients typically exhibit short stature and thin limbs due to poor growth and lipodystrophy. The distinctive facial features include mandibular hypoplasia, a beaked nose, prominent eyes, and dental crowding, resulting in a bird-like appearance. Common musculoskeletal abnormalities include joint contractures and spinal deformities such as kyphosis or scoliosis. Metabolic disturbances are also prevalent, including insulin resistance, diabetes mellitus, hepatomegaly with steatosis, and hypertriglyceridemia due to lipodystrophy [1–6].

The multisystemic nature of MDPL syndrome poses unique challenges for anesthetic management. However, to our knowledge, no reports have detailed the management of general anesthesia in these patients. In this report, we share our experience with general anesthesia in a patient with MDPL syndrome, aiming to provide insights that may guide safe and effective anesthetic practices for this vulnerable population. This case report adheres to the CARE (CAse REport) guidelines [7].

## Case presentation

A 15-year-old male (height 150.6 cm, body weight 35.6 kg, body mass index 15.7 kg/m^2^) with a confirmed diagnosis of MDPL syndrome, established through genetic testing, presented for laparoscopy and testicular extraction under general anesthesia due to testicular hypoplasia and cryptorchidism, both characteristic features of MDPL syndrome. The patient exhibited several hallmark features of MDPL syndrome, including mandibular hypoplasia, a small mouth, irregularly shaped teeth, a hooked nose, proptosis, scleroderma-like skin, hepatic steatosis, joint contractures, thin arms and legs with a wide trunk, and sensorineural deafness. His moderate hearing impairment was managed with a hearing aid, allowing for effective verbal communication. Blood tests revealed elevated liver enzymes, with aspartate aminotransferase at 79 U/L and alanine aminotransferase at 186 U/L, while serum triglyceride levels remained within the normal range. Although insulin resistance was present, diabetes mellitus was not. The chest X-ray was unremarkable, and an electrocardiogram showed a slightly prolonged Fridericia-corrected QT interval of 450 ms; however, there were no other cardiovascular issues. No cognitive dysfunction or intellectual disability was observed. Empagliflozin, a selective sodium-glucose cotransporter 2 inhibitor, was regularly administered but discontinued 3 days prior to surgery. This patient is the same individual previously reported by Okada et al. [8].

Upon entering the operating room, initial vital signs showed a non-invasive blood pressure of 136/90 mmHg, a heart rate of 82 bpm, and an oxygen saturation of 99%. Inserting a peripheral venous catheter into the forearm was challenging due to scleroderma-like skin but was successfully accomplished. After confirming a proper mask fit, preoxygenation with 6 L/min of oxygen was performed for 5 min before anesthesia induction. Thiamylal 150 mg and remifentanil 0.3 μg/kg/min were administered intravenously, followed by rocuronium 30 mg, after which mask ventilation was easily performed. Tracheal intubation was performed using a McGrath™ Mac video laryngoscope (Medtronic, Covidien Japan Ltd., Tokyo) with a 6.0-mm cuffed tracheal tube. The glottis was visualized by lifting the epiglottis with a size 3 laryngoscope blade, resulting in a Cormack and Lehane classification of grade 2b. Lid closure was difficult due to proptosis, so eye ointment and gauze were used to protect the corneas.

Anesthesia was maintained with 1.5% sevoflurane, a remifentanil infusion of 0.2–0.25 μg/kg/min, and 100 μg of fentanyl, ensuring stable vital signs. Acetaminophen 500 mg was administered for postoperative analgesia. After the surgery was completed, sugammadex 80 mg and naloxone 0.05 mg were given. The patient emerged from anesthesia without any issues, and there was no delayed awakening. The surgery lasted 70 min, with a total anesthesia time of 134 min. There were no complications, and the patient was discharged on the second postoperative day.

## Discussion

MDPL syndrome involves multisystemic abnormalities that require special consideration during general anesthesia. Key features include distinctive facial characteristics with mandibular hypoplasia, metabolic disturbances from lipodystrophy, and musculoskeletal abnormalities such as joint contractures. Through a comprehensive literature search of PubMed and SCOPUS databases, we identified 20 articles describing 38 cases of MDPL syndrome [1–3, 5, 6, 8–22]. The detailed search methodology is presented in Supplemental Material S1, while a summary of clinical features from previously reported cases is provided in Supplemental Material S2. Based on our case experience and a review of the literature, the anesthetic considerations for MDPL patients are summarized in Table 1. It is important to note that, as seen in our patient who did not have hypertriglyceridemia or diabetes mellitus, the severity of symptoms can vary among individuals with MDPL syndrome.

**Table 1:** Anesthetic considerations for patients with MDPL syndrome

Clinical features	Anesthetic considerations
Morphology	
✓ Short stature ✓ Bird-like facies including mandibular hypoplasia, small mouth, and dental overcrowding ✓ Loss of subcutaneous fat in the limbs ✓ Tight and thin skin/scleroderma-like skin/telangiectasias ✓ Prominent eyes	Advanced airway planning is essential. Careful selection of endotracheal tube size is necessary. Peripheral intravenous catheter insertion can be challenging. Fragile skin increases the risk of lip injuries during laryngoscopy and intubation. Apply eye ointment to protect the corneas, as patients may have difficulty closing their eyes
Metabolic profile	
✓ Insulin resistance ✓ Diabetes mellitus ✓ Hepatomegaly/hepatic steatosis ✓ Hypertriglyceridemia	Evaluation of diabetes-related complications and careful perioperative serum glucose management are essential. Liver dysfunction may alter drug metabolism. Elevated serum lipid levels require careful selection and use of anesthetic agents due to potential changes in the distribution of highly lipophilic intravenous anesthetics and delayed washout of volatile anesthetics. Prolonged total intravenous anesthesia with propofol may exacerbate elevated lipid levels, increasing the risk of complications
Musculoskeletal	
✓ Joint contractures ✓ Kyphosis/scoliosis	Careful positioning is crucial to avoid injury
Others	
✓ Sensorineural deafness	Consider visual or written communication methods and the use of hearing aids during awakening

*MDPL* mandibular hypoplasia, deafness, progeroid features, and lipodystrophy

Difficult airway management is a major concern for anesthesiologists, particularly due to the distinctive facial structure and mandibular dysplasia. In this case, successful intubation was achieved using a McGrath™ Mac video laryngoscope; however, the level of difficulty can vary depending on individual patient factors. Consequently, a comprehensive preoperative evaluation and advanced airway management planning are crucial. For determining the appropriate size of the endotracheal tube, it may be advisable to base the decision on the patient’s height or utilize ultrasound measurements of the subglottic area, as recommended in other progeroid disorders associated with short stature [23, 24].

Lipodystrophy leads to the absence of subcutaneous fat, hypertriglyceridemia, and hepatomegaly, which can significantly affect the pharmacokinetics and metabolism of anesthetics [25]. Serum lipid levels can impact the uptake and washout of volatile anesthetics, particularly those with a high blood/gas partition coefficient, potentially resulting in prolonged recovery times [25–27]. Total intravenous anesthesia with propofol should be used cautiously during long surgeries, as it could elevate blood triglyceride levels, increasing the risk of acute pancreatitis [23]. Lipophilic intravenous anesthetics like thiopental/thiamylal typically distribute first to central compartments, then to muscle, and finally to fat [28]. In patients with lipodystrophy, elevated serum lipids and the absence of normal fat stores can disrupt this typical distribution, potentially leading to unpredictable effects. Preoperative assessment of the patient’s lipid profile, including triglyceride levels, as well as liver function, is essential for anticipating pharmacokinetic challenges. In our patient, serum triglyceride levels were normal, allowing for anesthesia induction with thiamylal and maintenance with sevoflurane without any delays in recovery.

While MDPL syndrome shares some features with other progeroid disorders, it also has distinct characteristics [4, 19]. Patients with MDPL syndrome generally have a normal life expectancy and tend to live longer than those with other progeroid conditions [1, 19]. Unlike in Hutchinson-Gilford Progeria Syndrome or Werner Syndrome, cardiac or vascular issues such as severe atherosclerosis are less prominent in MDPL syndrome [17, 19]. Additionally, although POLD1 mutations have been identified in cancers such as colon cancer, there is no evidence suggesting an increased cancer risk in patients with MDPL syndrome [15, 19, 29]. Cognitive dysfunction and intellectual disability are also uncommon in this syndrome [17].

This report has the following strengths and limitations. Our described anesthetic management may provide practical guidance for anesthesiologists, addressing the current literature gap on general anesthesia in MDPL syndrome. Our comprehensive literature review can contribute to understanding this rare condition and its potential anesthetic risks. However, conclusions from a single case cannot be generalized to all MDPL cases. It should be noted that MDPL syndrome presents with a wide range of symptoms, which can vary among individuals.

In conclusion, MDPL syndrome presents multisystemic challenges that require careful preoperative assessment and planning for general anesthesia. Special attention is needed for airway management due to distinctive facial features and for the selection of anesthetic agents, considering the potential for altered pharmacokinetics associated with lipodystrophy.

## Supplementary Information

Supplementary Material 1. Supplementary material S1.

Supplementary Material 2. Supplementary material S2.

## Data Availability

Data sharing is not applicable to this case report.

## Abbreviation

MDPL syndrome: Mandibular hypoplasia, deafness, progeroid features, lipodystrophy
