# Supplementary material for: Anesthetic management of a patient with mandibular hypoplasia, deafness, progeroid features, lipodystrophy syndrome: a case report
Source: JA Clin Rep. 2024 Oct 10;10:64. doi: 10.1186/s40981-024-00747-8 (PMC11466922; doi:10.1186/s40981-024-00747-8)
Supplement: Supplementary file 1 — Supplementary Material 1. Supplementary material S1. [file 40981_2024_747_MOESM1_ESM.docx]

**Supplemental Material S1**

**Materials and Methods**

*Information Source and Search Strategy*

We conducted a comprehensive literature search using PubMed and SCOPUS databases for articles reporting cases of Mandibular hypoplasia, Deafness, Progeroid features, and Lipodystrophy (MDPL) syndrome on September 18, 2024. We used EndNote version 20.4 (Clarivate™, London, UK) to manage the retrieved literature.

*PubMed*

("mandibular hypoplasia"[All Fields] AND ("deafness"[MeSH Terms] OR "deafness"[All Fields] OR "deafnesses"[All Fields]) AND "progeroid"[All Fields] AND ("lipodystrophy"[MeSH Terms] OR "lipodystrophy"[All Fields] OR "lipodystrophies"[All Fields])) OR "mdpl"[All Fields]

*SCOPUS*

(TITLE-ABS-KEY ("mandibular hypoplasia" AND deafness AND progeroid AND lipodystrophy) OR TITLE-ABS-KEY (mdpl))

*Inclusion and Exclusion Criteria*

We included original articles, case reports, reviews, special articles, and letters describing original cases of MDPL syndrome. We excluded reports written in languages other than English and reports without full text availability. After removing duplicate entries in our initial screening, we reviewed titles and abstracts to exclude unrelated publications. Subsequently, full-text articles were assessed for eligibility. We also reviewed the references cited in articles that passed the second screening for additional relevant articles.

**Results**

The database search initially yielded 150 articles. After removing duplicates and applying the inclusion and exclusion criteria, we identified 18 articles. An additional two reports were identified through reference list review. In total, we included 20 articles describing 38 cases of MDPL syndrome. The following figure shows the flow diagram depicting the database search and literature selection process. Supplemental Material S2 summarizes the clinical features of cases included in this review.
